# Enhanced Inhibition of HNSCC Growth by α-Tomatine and Cisplatin via MAPK-Mediated Apoptosis

**DOI:** 10.3390/molecules31071084

**Published:** 2026-03-26

**Authors:** Ah-Reum Han, Hyeon-Ji Lim, Chang Hyun Jin, Ha-Yeon Song, Mi-Jeong Lee, Chan-Hun Jung

**Affiliations:** 1 Advanced Radiation Technology Institute, Korea Atomic Energy Research Institute (KAERI), Jeongeup-si 56212, Jeollabuk-do, Republic of Korea; arhan@kaeri.re.kr (A.-R.H.); chjin@kaeri.re.kr (C.H.J.); hysong93@kaeri.re.kr (H.-Y.S.); 2 Jeonju AgroBio-Materials Institute, Jeonju-si 54810, Jeollabuk-do, Republic of Korea; lhj0923@jami.re.kr; 3 Department of Human Nutrition, Food and Animal Sciences, College of Tropical Agriculture and Human Resilience, University of Hawai‘i at Mānoa, Honolulu, HI 96822, USA; leemj7@hawaii.edu

**Keywords:** α-tomatine, cisplatin, head and neck squamous cell carcinoma, apoptosis, combination therapy

## Abstract

Head and neck squamous cell carcinoma (HNSCC) is frequently associated with cisplatin resistance, which limits the therapeutic efficacy of conventional chemotherapy. In this study, we investigated whether α-tomatine could enhance cisplatin sensitivity and augment its antitumor efficacy in HNSCC cells. Treatment with 20 μM cisplatin alone induced relatively low cytotoxicity in FaDu and YD38 cells (18.45 ± 2.59% and 9.40 ± 2.33%, respectively). In contrast, co-treatment of FaDu and YD38 cells with cisplatin (20 μM) and a non-cytotoxic concentration of α-tomatine (2 μM) significantly increased cell death to 52.98 ± 7.84% and 40.40 ± 3.06%, respectively, compared with cisplatin monotherapy. The combination treatment markedly suppressed colony formation, indicating reduced clonogenic survival, and significantly enhanced apoptosis through the simultaneous activation of intrinsic and extrinsic apoptotic pathways. The enhanced apoptosis was driven by the activation of the mitogen-activated protein kinase (MAPK) signaling cascade. Furthermore, the enhanced antitumor effect of α-tomatine and cisplatin was confirmed in a xenograft tumor model. These findings demonstrate that α-tomatine enhances cisplatin-induced apoptosis via MAPK-mediated signaling, supporting its role as a chemosensitizing agent for HNSCC.

## 1. Introduction

Head and neck squamous cell carcinoma (HNSCC), which develops in the pharynx, larynx, and oral cavity, account for roughly 600,000 annual diagnoses worldwide, making it the sixth most common cancer [1]. Key risk factors include viral infection, heavy alcohol use, and tobacco consumption [2]. While the standard of care typically involves surgical resection and radiotherapy, less invasive approaches like chemotherapy are frequently utilized to preserve oral function and patient aesthetics [3,4]. Cisplatin stands as the standard for HNSCC chemotherapy. In patients with locally advanced diseases, initial response rates can reach 50%; however, the remaining half often show no response or eventually develop acquired resistance. Such resistance is a primary driver of treatment failure and disease recurrence [5]. Consequently, oncology research is moving toward combination therapies to achieve synergic outcomes and mitigate the side effects associated with high-dose cisplatin [6]. Recent evidence suggests that certain low-toxicity natural compounds can significantly bolster the efficacy of platinum-based agents [7,8,9,10].

α-Tomatine is a steroid glycoalkaloid found in the immature fruits of *Lycopersicon esculentum* (green tomatoes). It has demonstrated a wide array of biological properties, including anti-inflammatory, antibacterial, anti-obesity, neuroprotective, muscle strengthening, and anticancer effects [11]. While the cancer-preventive reputation of tomatoes is often attributed to the antioxidant lycopene, glycoalkaloids like α-tomatine have shown independent potency in suppressing tumor growth and inducing apoptosis [12]. Although preliminary studies indicate that combining this tomato alkaloid with cisplatin can trigger apoptosis in acute myeloid leukemia [13], its potential in HNSCC has not been established. Therefore, this study aimed to evaluate the potential enhanced antitumor effects of α-tomatine and cisplatin in HNSCC cell lines and a xenograft tumor model, as well as underlying molecular mechanisms.

## 2. Results

### 2.1. α-Tomatine Enhances Cisplatin-Induced Cytotoxicity in HNSCC

**Figure 1:**
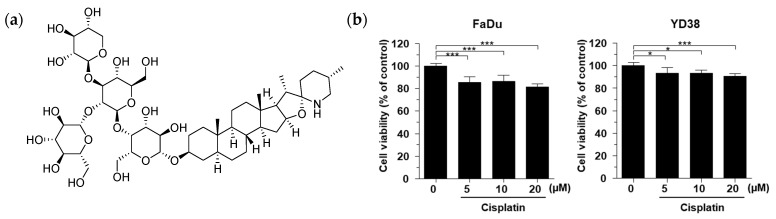

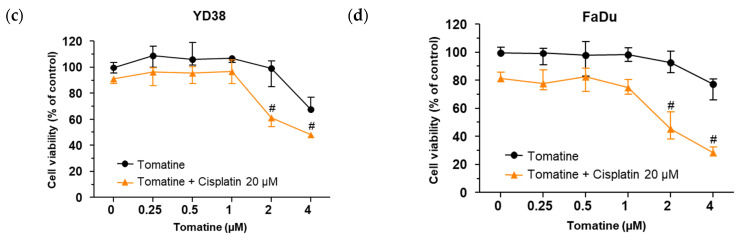
Combined treatment with α-tomatine and cisplatin enhances cytotoxic effect in HNSCC. (**a**) Structure of α-tomatine. (**b**) Cytotoxic effect of cisplatin in FaDu and YD38 cells. (**c**,**d**) Cytotoxic effects of combined α-tomatine and cisplatin in FaDu and YD38 cells. Data are expressed as mean ± SD (*n* = 3). Statistical significance was determined by one-way ANOVA followed by Tukey’s post hoc test. * *p* < 0.05, and *** *p* < 0.001; # *p* < 0.05 vs. cisplatin (20 μM) only group.

The cytotoxic effects of α-tomatine in combination with cisplatin were assessed in HNSCC cell lines using the MTT assay. To establish baseline sensitivity and determine an optimal dose for combination studies, FaDu and YD38 cells were initially treated with cisplatin monotherapy at concentrations of 5, 10, and 20 μM for 24 h, rather than determining IC_50_ values (Figure 1b). As shown in Figure 1b, cisplatin monotherapy at 20 μM resulted in limited cytotoxicity, reducing cell viability by 18.45 ± 2.59% in FaDu cells and 9.40 ± 2.33% in YD38 cells. Based on these screening results, 20 μM cisplatin was selected as a sensitizing dose to evaluate the potential enhanced effects of α-tomatine. Furthermore, to evaluate the sensitizing potential of α-tomatine within a biologically optimized rage that could enhance the cytotoxic efficacy of the selected 20 μM cisplatin, the enhanced effects of the combination treatment were assessed using increasing concentration of α-tomatine (0.25, 0.5, 1, 2, and 4 μM). In YD38 cells, co-treatment with 2 μM and 4 μM α-tomatine significantly enhanced cisplatin-induced cytotoxicity, reducing cell viability by 40.40 ± 3.06% and 51.86 ± 0.96%, respectively (Figure 1c). A more pronounced reduction in cell viability was observed in FaDu cells, where combined treatment with 20 μM cisplatin and 2, or 4 μM α-tomatine decreased cell viability by 52.98 ± 7.84% and 71.02 ± 1.90%, respectively (Figure 1d). These results indicate that α-tomatine significantly enhances cisplatin-induced cytotoxicity in HNSCC cells in a dose-dependent manner by further reducing the viability beyond the level achieved by cisplatin monotherapy alone.

### 2.2. Combined Treatment with α-Tomatine and Cisplatin Suppresses Proliferation and Induces Apoptosis

**Figure 2:**
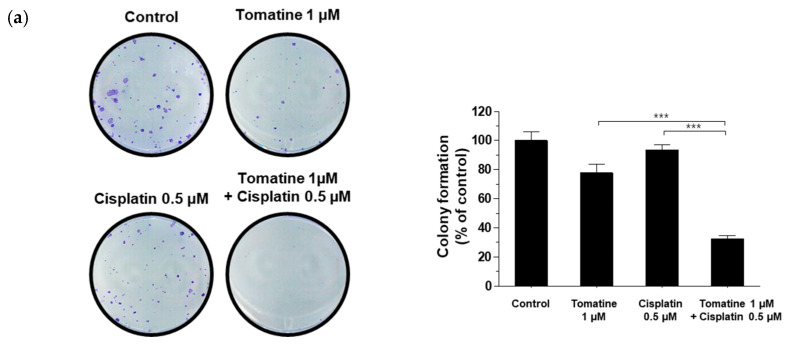

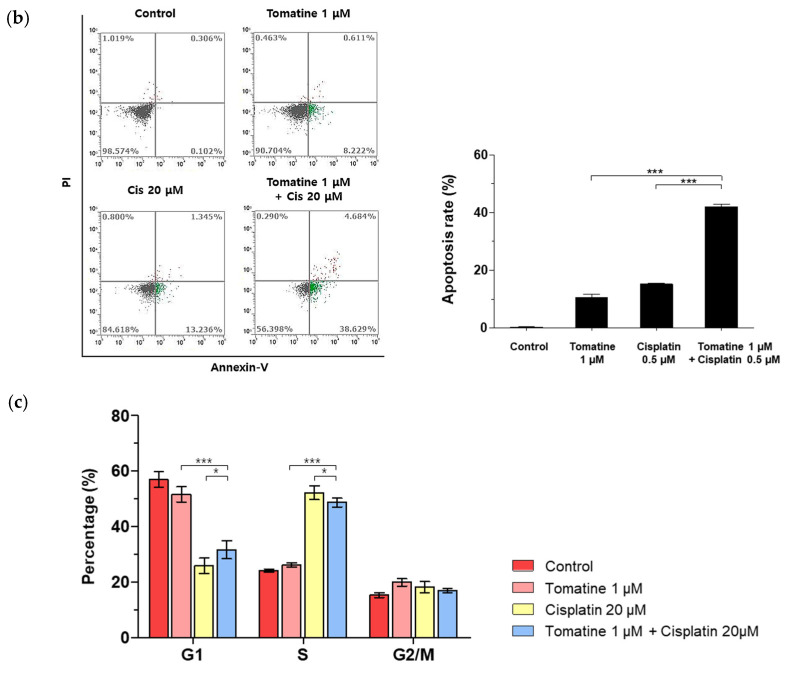
Combined treatment with α-tomatine and cisplatin regulates proliferation and apoptosis in HNSCC. (**a**) Colony formation assay. (**b**) Flow cytometry. (**c**) Cell cycle arrest. Data are expressed as mean ± SD (*n* = 3). Statistical significance was determined by one-way ANOVA followed by Tukey’s post hoc test. * *p* < 0.05, and *** *p* < 0.001.

To assess the long-term effects of α-tomatine and cisplatin on proliferative capacity, a colony formation assay was performed using FaDu cells. In this assay, cisplatin was used at a lower concentration of 0.5 μM to account for its cumulative toxicity over the extended treatment period. As shown in Figure 2a, treatment with 1 μM α-tomatine or 0.5 μM cisplatin alone resulted in modest reductions in colony formation (22.27 ± 5.87% and 6.21 ± 3.17%, respectively). In contrast, combined treatment markedly suppressed colony growth by 67.45 ± 2.21% (Figure 2a). Apoptotic cell death was further evaluated by Annexin V-FITC/PI staining. Co-treatment with 1 μM α-tomatine and 20 μM cisplatin significantly increased the proportion of apoptotic cells to 41.91 ± 1.71%, compared with 10.69 ± 1.98% and 15.25 ± 0.59% observed following treatment with α-tomatine or cisplatin alone, respectively (Figure 2b). These results indicate that the therapeutic outcome of the combined treatment is an enhancement rather than a summation effect. Cell cycle distribution was analyzed by flow cytometry. While tomatine alone did not have significant effects on the cell cycle distribution, cisplatin monotherapy increased the proportion of cells in the S phase; however, combination treatment significantly increased the G1-phase population while reducing the S-phase fraction (Figure 2c). These findings indicate that the combined treatment of α-tomatine and cisplatin reduces clonogenic survival, enhances apoptosis, and alters cell cycle distribution in FaDu cells.

### 2.3. Combined Treatment Activates Both Intrinsic and Extrinsic Apoptotic Pathways

**Figure 3:**
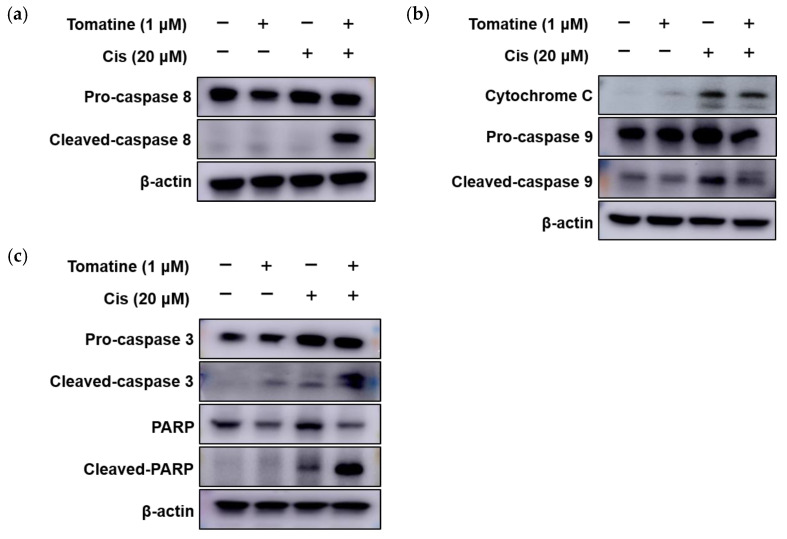
Combined treatment with α-tomatine and cisplatin significantly enhanced apoptosis induction via both intrinsic and extrinsic pathways in FaDu cells. (**a**) Expression levels of the extrinsic apoptosis marker, caspase 8; (**b**) Intrinsic apoptosis markers, cytochrome C and caspase 9; and (**c**) Execution-phase apoptosis markers, caspase 3 and PARP, were analyzed by Western blotting.

To determine whether apoptosis induced by α-tomatine and cisplatin involved the extrinsic (death receptor-mediated) and/or intrinsic (mitochondrial) pathways, protein expression levels of key apoptotic markers were examined by Western blot analysis. Cisplatin and α-tomatine monotherapy did not induce detectable cleavage of caspase-8, an initiator of the extrinsic apoptotic pathway; however, cleaved caspase-8 levels were markedly increased following combination treatment (Figure 3a). In contrast, cisplatin alone increased the expression of intrinsic apoptotic markers, including cytochrome c and cleaved caspase-9, whereas their levels were slightly reduced in cells treated with the combination of α-tomatine and cisplatin (Figure 3b). Notably, the expression of downstream executioner proteins, including cleaved caspase-3 and cleaved poly (ADP-ribose) polymerase (PARP), was significantly elevated in the combination-treated group compared with cisplatin monotherapy (Figure 3c). These data indicate that the addition of α-tomatine to cisplatin treatment is associated with the activation of both extrinsic and intrinsic apoptotic signaling pathways, leading to enhanced executioner caspase activation.

### 2.4. Combined Treatment Enhances MAPK Signaling Activation in FaDu Cells

**Figure 4:**
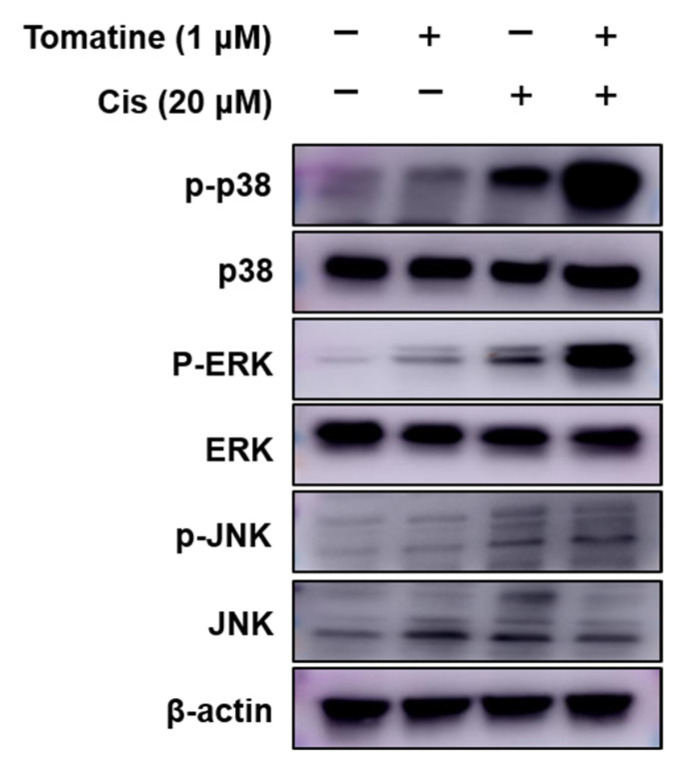
Combined treatment with α-tomatine and cisplatin significantly enhanced the activation of the MAPK signaling pathway in FaDu cells. Western blotting was performed to evaluate both phosphorylation and total forms of key MAPK pathway proteins, including p38, ERK, and JNK.

To examine the involvement of the mitogen-activated protein kinase (MAPK) signaling pathway in the combined effects of α-tomatine and cisplatin, the phosphorylation status of p38, ERK, and JNK was analyzed in FaDu cells. Co-treatment with 1 μM α-tomatine and 20 μM cisplatin resulted in a marked increase in the phosphorylation levels of p38, ERK, and JNK compared with those observed following treatment with either agent alone (Figure 4). These results indicate that combined α-tomatine and cisplatin treatment is associated with enhanced activation of MAPK signaling in FaDu cells.

### 2.5. Combined α-Tomatine and Cisplatin Treatment Suppresses Tumor Growth in a Xenograft Model

**Figure 5:**
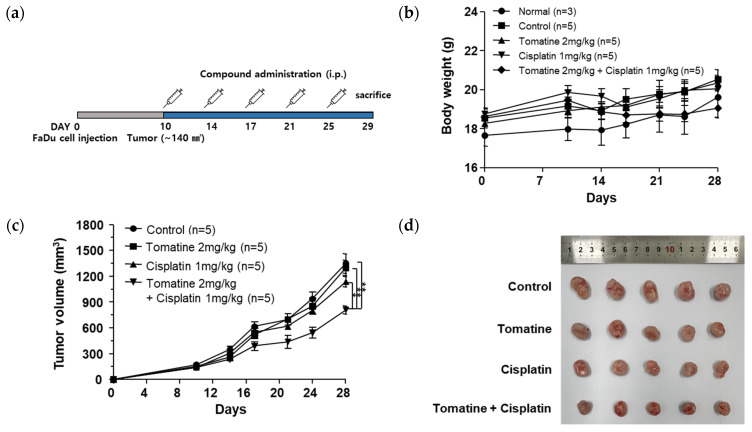
Combined treatment with α-tomatine and cisplatin significantly enhanced anticancer effects in xenograft tumor model. (**a**) Schematic representation. (**b**) Changes in body weight and (**c**) tumor volume during the experimental period. (**d**) Representative tumor images from each group at the endpoint. Normal, Normal control group; Control, xenograft tumor-bearing group; Tomatine, 2 mg/kg α-tomatine-treated group; Cisplatin, 1 mg/kg cisplatin-treated group, Tomatine + Cisplatin, combination treated group (2 mg/kg α-tomatine + 1 mg/kg cisplatin). Data are expressed as mean ± SD (*n* = 5 per group). Statistical significance was determined by one-way ANOVA followed by Tukey’s post hoc test. * *p* < 0.05, and ** *p* < 0.01.

To assess whether the in vitro findings were recapitulated in vivo, a xenograft model was established in BALB/c nude mice. Once tumors reached an average volume of approximately 140 mm^3^, mice were administered intraperitoneal injections of α-tomatine (2 mg/kg) and/or cisplatin (1 mg/kg) for a total of five times over a 19-day period (Figure 5a). No significant changes in body weight were observed among the treatment groups throughout the experimental period, indicating that the treatments were well tolerated (Figure 5b). At the end of the study, tumor volumes in the α-tomatine (1301.53 ± 194.50 mm^3^) and cisplatin (1138.83 ± 104.49 mm^3^) monotherapy groups were not significantly different from those in the control group (1352.55 ± 251.82 mm^3^). In contrast, mice receiving the combination treatment exhibited a significant reduction in tumor volume (811.07 ± 104.49 mm^3^) compared with the control and monotherapy groups (Figure 5c,d). These results demonstrate that combined α-tomatine and cisplatin treatment more effectively inhibits tumor growth in vivo than either agent alone, without apparent systemic toxicity.

## 3. Discussion

Cisplatin remains a cornerstone chemotherapeutic agent for a wide range of malignancies, including hematologic cancers such as leukemia and lymphoma, as well as solid tumors of the ovary, lung, bladder, and head and neck [14,15,16,17]. However, its clinical effectiveness is frequently compromised by the development of drug resistance and dose-limiting toxicities [18,19]. To overcome these limitations, increasing attention has been directed toward the use of natural compounds as adjuvant agents to enhance the therapeutic efficacy of cisplatin while minimizing systemic toxicity [20,21].

α-Tomatine, a steroid glycoalkaloid derived from green tomatoes, has demonstrated potent antitumor properties as a monotherapy across various models, including malignant melanoma, hepatocellular carcinoma, prostate cancer, and lung cancer [22,23,24,25]. Mechanistically, α-tomatine has been shown to inhibit angiogenesis in metastatic melanoma [22], modulate p53- and reactive oxygen species-mediated signaling in hepatocellular carcinoma [23], and suppress NF-κB activation in prostate and lung cancer cells [24,25]. In addition, a recent study demonstrated that tomatidine, the aglycone form of α-tomatine, sensitizes acute myeloid leukemia cells to cisplatin by enhancing apoptosis through modulation of PARP and caspase-3 activity [13]. From a structural perspective, α-tomatine is a type of steroid saponins composed of a steroid backbone (tomatidine) and a sugar moiety (lycotetraose). Steroid saponins are bioactive compounds with surfactant-like properties, known to interact with cholesterol in cell membranes to increase membrane permeability or induce structural changes [26]. These membrane-mediated effects are associated with potent biological activities, including anticancer, anti-inflammatory, and immunomodulatory effects [27]. Thus, the combination of a lipophilic steroidal nucleus and a hydrophilic carbohydrate chain consider α-tomatine to effectively interact with cellular targets and membranes, potentially promoting cellular uptake or efficacy of combination drugs. While these findings provided an initial rationale for combining tomato-derived alkaloids with platinum-based agents, the potential relevance of this approach in solid epithelial malignancies, particularly HNSCC, had not been fully explored.

In the present study, we demonstrate that α-tomatine enhances cisplatin-induced cytotoxicity in HNSCC cell lines, including FaDu and YD38 cells. The combination treatment resulted in greater growth inhibition and apoptotic induction than either agent alone, supporting the role of α-tomatine as a chemosensitizing agent in this cellular context.

To further elucidate the mechanisms underlying this interaction, we examined key pathways involved in cell cycle regulation and programmed cell death. Although both α-tomatine and cisplatin are known to inhibit cell proliferation and induce apoptosis, they preferentially affect distinct phases of the cell cycle [24,28]. In the present study, we observed that α-tomatine primarily promoted G1-phase arrest, whereas cisplatin increased the proportion of cells in the S phase. Notably, co-treatment shifted the cell cycle distribution toward a G1 phase-predominant arrest, reversing the cisplatin-induced accumulation of S-phase cells and correlating with enhanced growth inhibition and apoptotic induction compared with cisplatin monotherapy.

Programmed cell death in cancer cells can proceed through multiple pathways, including apoptosis, necroptosis, and autophagy-associated cell death [29,30]. However, in this study, we focused on apoptotic cell death based on several definitive biochemical and cytometric indicators, such as Annexin V/PI staining and activation of the caspase cascade [31]. Annexin V/PI staining revealed a significant increase in apoptotic cell populations following combined treatment with α-tomatine and cisplatin (Figure 2b). In parallel, we observed activation of the caspase cascade, including increased cleavage of caspase-8, caspase-9, caspase-3, and PARP (Figure 3). These results indicate that the combination of α-tomatine and cisplatin activates both intrinsic and extrinsic apoptotic pathways. While cisplatin predominantly engages the mitochondrial intrinsic pathway, the addition of α-tomatine was associated with activation of the extrinsic, death receptor-mediated pathway, as evidenced by increased cleavage of caspase-8. The concurrent engagement of these pathways culminated in enhanced activation of downstream executioner proteins, including cleaved caspase-3 and cleaved PARP. However, as markers of other programmed cell death pathways were not directly examined in this study, we cannot completely exclude the possible involvement of additional cell death mechanisms. Future studies incorporating pathway-specific analyses would more clearly elucidate the relative contributions of various cell death mechanisms to the overall antitumor effect.

In HNSCC, the MAPK pathway exhibits a dual role, often shifting from pro-survival to pro-apoptotic signaling under severe cellular stress [32]. In the present study, the marked phosphorylation of JNK, p38, and ERK induced by α-tomatine and cisplatin closely correlated with the activation of caspase-3 and PARP. These findings suggest that the combination therapy engages the MAPK cascade as a stress-responsive mediator to drive apoptosis, consistent with previous reports that sustained activation of JNK and p38 sensitizes HNSCC cells to cisplatin-induced apoptosis [19,33]. Although pharmacological inhibition studies would be required for definitive mechanistic validation, the consistent association between MAPK activation and apoptotic markers observed in this study, in line with previous reports, suggests that MAPK signaling likely contributes to the enhanced chemosensitivity.

Finally, the enhanced antitumor potential of the combination therapy was validated in a xenograft model to confirm our in vitro findings at the systemic level. While monotherapy with either α-tomatine or cisplatin at the tested doses failed to significantly reduce tumor volumes compared to the control group, their combined administration led to a marked suppression of tumor growth. These results further highlight the potential of α-tomatine as a promising adjuvant therapy to enhance the therapeutic efficacy of cisplatin in the treatment of head and neck cancer.

In summary, the current study demonstrates that α-tomatine effectively facilitates apoptosis in response to both sublethal DNA damage (at low cisplatin doses) and acute genomic stress (at high cisplatin doses). By simultaneously activating the extrinsic pathway, a signaling route typically underutilized by cisplatin monotherapy, α-tomatine lowers the apoptotic trigger threshold in HNSCC cells. Consequently, we propose a mechanistic model in which α-tomatine serves as a potent chemosensitizer that ensures committed cell death across a broad range of cisplatin concentrations, providing a robust rationale for its clinical potential as an adjunctive therapy to enhance cisplatin efficacy and address chemoresistance in HNSCC.

## 4. Materials and Methods

### 4.1. Reagents and Antibodies

Cisplatin, crystal violet, cremophor, glucose and dimethyl sulfoxide (DMSO) were obtained from Sigma-Aldrich (Sigma-Aldrich, St. Louis, MO, USA). α-Tomatine was supplied by PhytoLab (PhytoLab, Vestenbergsgreuth, Germany). Stock solutions (10 mM) of α-Tomatine and cisplatin were prepared separately by dissolving each compound in DMSO. To avoid potential chemical interactions or degradation, both compounds were individually and freshly diluted in the culture medium immediately before each experiment. Culture media, including Minimum Essential Medium (MEM) and RPMI-1640, supplemented with fetal bovine serum (FBS), were obtained from HyClone (HyClone, Logan, UT, USA). Cell detachment and washing were performed using 0.25% Trypsin-EDTA and phosphate-buffered Saline (PBS), which were purchased from Gibco (Gibco, Gaithersburg, MD, USA). For experimental assays, the EZ-Cytox Cell Viability Assay Kit was purchased from DOGEN (DOGEN, Seoul, Republic of Korea). The Annexin V-FITC/PI apoptosis detection kit and Vybrant^®^ DyeCycle™ Green Stain were acquired from Invitrogen (Invitrogen, Carlsbad, CA, USA). Protein analysis tools, including the BCA Protein Assay kit and PVDF membrane, were from Bio-Rad (Bio-Rad, Hercules, CA, USA). Matrigel^®^ Matrix was supplied by Corning (Corning, NY, USA). Primary antibodies against PARP, cleaved-PARP, various caspases (pro- and cleaved forms of caspase-3, caspase-8, and caspase-9), cytochrome c, and members of the MAPK pathway (p38, ERK, JNK, and their phosphorylated forms), as well as HRP-labeled secondary antibodies and β-actin, were all purchased from Cell Signaling Technology (Cell Signaling Technology, Danvers, MA, USA).

### 4.2. Maintenance and Experimental Conditions

Human HNSCC cell lines FaDu and YD38 were obtained from the Korean Cell Line Bank (KCLB, Seoul, Republic of Korea). FaDu cells were grown in MEM, and YD38 cells were cultured in RPMI-1640, each supplemented with 10% FBS. All cell cultures were incubated at 37 °C in a humidified atmosphere containing 5% CO_2_.

### 4.3. Evaluation of Cell Viability

Cells were seeded into 96-well plates at a density of 1 × 10^4^ cells per well and stabilized for 24 h to treatment. They were then exposed to cisplatin alone (5, 10, or 20 μM) or co-treated with cisplatin (20 μM) in combination with increasing concentrations of α-tomatine (0, 0.25, 0.5, 1, 2, or 4 μM) for an additional 24 h. Cell viability was quantified using the EZ-Cytox assay, and absorbance was measured at 450 nm using a microplate spectrophotometer (Thermo Fisher Scientific, Waltham, MA, USA).

### 4.4. Proliferation Assessment via Colony Formation

FaDu cells were seeded into 6-well plates at a density of 2 × 10^2^ cells per well to ensure the formation of discrete, quantifiable colonies. After 24 h of growth, the medium was replaced with fresh media containing α-tomatine (1 μM), cisplatin (0.5 μM), or the combination treatment for an 8 day incubation period. The resulting colonies were fixed with 0.2% crystal violet, washed, and quantified. All experimental procedures were performed in triplicate to ensure reproducibility and statistical validity.

### 4.5. Apoptosis and Cell Cycle Analysis

To measure apoptosis, FaDu cells were plated at a density of 2 × 10^5^ cells per well and exposed to α-tomatine (1 μM), cisplatin (0.5 μM), or their combination for 24 h. Apoptosis was assessed by FITC Annexin V-FITC/Dead Cell Apoptosis Kit according to the manufacturer’s protocol. Treated cells were collected, stained with FITC Annexin V and propidium iodide (PI) and subsequently analyzed by flow cytometry (Thermo Fisher Scientific). For cell cycle distribution, treated cells were stained with Vybrant^®^ DyeCycle™ Green (10 μM) at 37 °C for 30 min and subsequently analyzed by flow cytometry at 488 nm.

### 4.6. Western Blot Analysis

FaDu cells were harvested and lysed on ice using RIPA buffer. After centrifugation, protein levels were quantified using the BCA Kit. Equivalent amounts of protein were separated via SDS-PAGE (12%) and subsequently transferred onto PVDF membranes. Following a blocking step, membranes were incubated with primary antibodies overnight at 4 °C, then treated with HRP-linked secondary antibodies for 1 h at room temperature. Immunoreactive signals were detected using an Amershan Imager 600 system (GE HealthCare, Chicago, IL, USA).

### 4.7. In Vivo Xenograft Studies

The animal study was designed and reported in accordance with the ARRIVE Essential 10 guidelines to ensure reproducibility and transparency. A single mouse was defined as the experimental unit for all analyses. Five-week-old female specific pathogen-free (SPF) Balb/c nude mice (*n* = 23, 15–17 g) were acclimated for one week under controlled laboratory conditions (22 ± 2 °C, 55 ± 5% humidity, 12 h light/dark cycle) prior to the experiment. To establish the HNSCC xenograft model, FaDu cells (1 × 10^6^ cells in 100 μL; PBS:Matrigel = 1:1) were injected subcutaneously into the right flank of each mouse. Once tumors reached approximately 140 mm^3^ (day 10), all tumor-bearing mice were randomly assigned to the experimental groups. This randomization excluded the normal control group (*n* = 3, no tumor induction). Consistent with our previously established xenograft tumor studies, each experimental group was composed of five mice [34]. The remaining mice were allocated to four groups: (1) Tumor control (*n* = 5, vehicle only), (2) α-tomatine (*n* = 5, 2 mg/kg), (3) Cisplatin (*n* = 5, 1 mg/kg), and (4) Combination (*n* = 5, 2 mg/kg α-tomatine + 1 mg/kg cisplatin).

The dosages for this combination therapy were determined based on literature reviews of single-agent treatments, as no previous reports on the combined in vivo administration of these compounds were available. Standard effective antitumor dosages in xenograft models were identified as approximately 3 mg/kg for cisplatin and 5 mg/kg for α-tomatine [35,36]. To specifically evaluate the sensitizing capacity of α-tomatine against sub-lethal levels of cisplatin while minimizing systemic toxicity, the cisplatin dosage was lowered to 1 mg/kg. Based on molecular weight characteristics and literature-derived ratios, the α-tomatine dosage was optimized to 2 mg/kg to establish a robust and well-tolerated therapeutic range.

To minimize social interaction across groups, animals belonging to the same experimental group were housed together in the same cage. All 23 animals were accounted for in the final results, and no data points were excluded during the study period. While group allocation was known to the researchers during treatment, tumor measurements were conducted using a standardized, objective protocol to minimize assessment bias.

The primary outcome measure was the tumor volume, and the secondary outcome was body weight to monitor systemic toxicity. Notably, the normal control group was excluded from all statistical analyses except for the body weight evaluation. The stability of body weight monitored throughout the treatment period confirmed the safety of the selected dosages. For drug formulation, α-tomatine was dissolved in a vehicle consisting of 5% DMSO, 5% Cremophor, and 90% D5W (5% glucose). Notably, to minimize potential vehicle-related systemic toxicity, the final concentration of DMSO was strictly maintained below 1% (*v*/*v*) in all administered solutions. Cisplatin was dissolved in sterile 0.9% saline. In the combination group, to prevent any potential chemical interaction or degradation prior to systemic circulation, α-tomatine and cisplatin were administered through two separate intraperitoneal (i.p.) injections.

Treatments were administered via intraperitoneal (i.p.) injection a total of five times over a 19-day period. Tumor growth and body weight were monitored twice per week, with volumes (V) calculated using the formula: V = (length × width^2^)/2. All experimental procedures received ethical approval from the Institutional Animal Care and Use Committee of the Jeonju AgroBio-Mater Institute (JAMI IACUC 2025002).

### 4.8. Data Processing and Statistical Analysis

All experimental data are expressed as the mean ± standard deviation (SD). Statistical analyses were performed using GraphPad Prism software (version 5.01; GraphPad Software, San Diego, CA, USA). Significant differences between groups were determined using one-way analysis of variance (ANOVA), followed by Tukey’s post hoc test for multiple comparisons. A *p*-value of less than 0.05 was considered the threshold for statistical significance.

## Data Availability

The data presented in this study are available upon request from the corresponding authors.

## References

[1] Leemans C.R., Braakhuis B.J., Brakenhoff R.H. (2011). The molecular biology of head and neck cancer. Nat. Rev. Cancer.

[2] Pezzuto F., Buonaguro L., Caponigro F., Ionna F., Starita N., Annunziata C., Buonaguro F.M., Tornesello M.L. (2015). Update on head and neck cancer: Current knowledge on epidemiology, risk factors, molecular features and novel therapies. Oncology.

[3] Mordzińska-Rak A., Telejko I., Adamczuk G., Trombik T., Stepulak A., Błaszczak E. (2025). Advancing Head and Neck Cancer Therapies: From Conventional Treatments to Emerging Strategies. Biomedicines.

[4] Wang Y., Han J., Zhu Y., Huang N., Qu N. (2025). New advances in the therapeutic strategy of head and neck squamous cell carcinoma: A review of latest therapies and cutting-edge research. Biochim. Biophys. Acta-Rev. Cancer.

[5] Shen D.W., Pouliot L.M., Hall M.D., Gottesman M.M. (2012). Cisplatin resistance: A cellular self-defense mechanism resulting from multiple epigenetic and genetic changes. Pharmacol. Rev..

[6] Fan W., Yung B., Huang P., Chen X. (2017). Nanotechnology for multimodal synergistic cancer therapy. Chem. Rev..

[7] Kanai M., Hatano E., Kobayashi S., Fujiwara Y., Marubashi S., Miyamoto A., Shiomi H., Kubo S., Ikuta S., Yanagimoto H. (2015). A multi-institution phase II study of gemcitabine/cisplatin/S-1 (GCS) combination chemotherapy for patients with advanced biliary tract cancer (KHBO 1002). Cancer Chemother. Pharmacol..

[8] Choi H.S., Kim Y.K., Yun P.Y. (2021). Cisplatin plus cetuximab inhibits cisplatin-resistant human oral squamous cell carcinoma cell migration and proliferation but does not enhance apoptosis. Int. J. Mol. Sci..

[9] Al Fayi M., Otifi H., Alshyarba M., Dera A.A., Rajagopalan P. (2020). Thymoquinone and curcumin combination protects cisplatin-induced kidney injury, nephrotoxicity by attenuating NFκB, KIM-1 and ameliorating Nrf2/HO-1 signalling. J. Drug Target..

[10] Lin M., Pan C., Xu W., Li J., Zhu X. (2020). Leonurine promotes cisplatin sensitivity in human cervical cancer cells through increasing apoptosis and inhibiting drug-resistant proteins. Drug Des. Dev. Ther..

[11] Wang L.H., Tan D.H., Zhong X.S., Jia M.Q., Ke X., Zhang Y.M., Cui T., Shi L. (2024). Review on toxicology and activity of tomato glycoalkaloids in immature tomatoes. Food Chem..

[12] Dey P., Kundu A., Chakraborty H.J., Kar B., Choi W.S., Lee B.M., Bhakta T., Atanasov A.G., Kim H.S. (2019). Therapeutic value of steroidal alkaloids in cancer: Current trends and future perspectives. Int. J. Cancer.

[13] Ayvaz H.B., Yenigül M., Gencer Akçok E.B. (2024). Tomatidine, a Steroidal Alkaloid, Synergizes with Cisplatin to Inhibit Cell Viability and Induce Cell Death Selectively on FLT3-ITD+ Acute Myeloid Leukemia Cells. Cell Biochem. Biophys..

[14] Yu X., Jia L., Tang Q., Zhou Q., Wang G., Wang S. (2025). Regulation of cisplatin resistance in lung cancer by epigenetic mechanisms. Clin. Epigenetics.

[15] Tsvetkova D., Ivanova S. (2022). Application of Approved Cisplatin Derivatives in Combination Therapy against Different Cancer Diseases. Molecules.

[16] Beheshtizadeh N., Kolahi Azar H., Seraji A.A., Zarei M., Hajian Monfared M., Mahheidari N., Darghiasi S.F., Afandideh F., Badihi E., Tabatabaei S.Z. (2025). Cancer-affected tissue regeneration employing cisplatin-loaded polymeric nanoplatforms. Biomed. Pharmacother..

[17] Brown A., Kumar S., Tchounwou P.B. (2019). Cisplatin-Based Chemotherapy of Human Cancers. J. Cancer Sci. Ther..

[18] Romani A.M.P. (2022). Cisplatin in cancer treatment. Biochem. Pharmacol..

[19] Roy S., Kar M., Roy S., Saha A., Padhi S., Banerjee B. (2018). Role of β-catenin in cisplatin resistance, relapse and prognosis of head and neck squamous cell carcinoma. Cell. Oncol..

[20] Dasari S., Njiki S., Mbemi A., Yedjou C.G., Tchounwou P.B. (2022). Pharmacological Effects of Cisplatin Combination with Natural Products in Cancer Chemotherapy. Int. J. Mol. Sci..

[21] Rajendran G., Taylor J.A., Woolbright B.L. (2021). Natural products as a means of overcoming cisplatin chemoresistance in bladder cancer. Cancer Drug Resist..

[22] Serratì S., Porcelli L., Guida S., Ferretta A., Iacobazzi R.M., Cocco T., Maida I., Tamasi G., Rossi C., Manganelli M. (2020). Tomatine Displays Antitumor Potential in In Vitro Models of Metastatic Melanoma. Int. J. Mol. Sci..

[23] Echeverría C., Martin A., Simon F., Salas C.O., Nazal M., Varela D., Pérez-Castro R.A., Santibanez J.F., Valdés-Valdés R.O., Forero-Doria O. (2022). In Vivo and in vitro antitumor activity of tomatine in hepatocellular carcinoma. Front. Pharmacol..

[24] Lee S.T., Wong P.F., Cheah S.C., Mustafa M.R. (2011). Alpha-tomatine induces apoptosis and inhibits nuclear factor-kappa B activation on human prostatic adenocarcinoma PC-3 cells. PLoS ONE.

[25] Shieh J.M., Cheng T.H., Shi M.D., Wu P.F., Chen Y., Ko S.C., Shih Y.W. (2011). α-Tomatine suppresses invasion and migration of human non-small cell lung cancer NCI-H460 cells through inactivating FAK/PI3K/Akt signaling pathway and reducing binding activity of NF-κB. Cell Biochem. Biophys..

[26] Frenkel N., Makky A., Sudji I.R., Wink M., Tanaka M. (2014). Mechanistic investigation of interactions between steroidal saponin digitonin and cell membrane models. J. Phys. Chem. B.

[27] Cui A., Liu H., Liu X., Zhang M., Xiao B., Wang B., Yang J. (2024). Steroidal saponins: Natural compounds with the potential to reverse tumor drug resistance (Review). Oncol. Lett..

[28] Jafarzadeh E., Montazeri V., Aliebrahimi S., Sezavar A.H., Ghahremani M.H., Ostad S.N. (2025). Targeting Cancer Stem Cells and Hedgehog Pathway: Enhancing Cisplatin Efficacy in Ovarian Cancer with Metformin. J. Cell Mol. Med..

[29] Ouyang L., Shi Z., Zhao S., Wang F.T., Zhou T.T., Liu B., Bao J.K. (2012). Programmed cell death pathways in cancer: A review of apoptosis, autophagy and programmed necrosis. Cell Prolif..

[30] Dho S.H., Cho M., Woo W., Jeong S., Kim L.K. (2025). Caspases as master regulators of programmed cell death: Apoptosis, pyroptosis and beyond. Exp. Mol. Med..

[31] Kaur A., Pandey R.K., Mehrotra S. (2025). Evolving methodologies for identification and differentiation of regulated cell death modalities. Prog. Mol. Biol. Transl. Sci..

[32] Yue J., López J.M. (2020). Understanding MAPK Signaling Pathways in Apoptosis. Int. J. Mol. Sci..

[33] Mansouri A., Ridgway L.D., Korapati A.L., Zhang Q., Tian L., Wang Y., Siddik Z.H., Mills G.B., Claret F.X. (2003). Sustained activation of JNK/p38 MAPK pathways in response to cisplatin leads to Fas ligand induction and cell death in ovarian carcinoma cells. J. Biol. Chem..

[34] Kim Y.R., Han A.R., Kim J.B., Jung C.H. (2021). Dendrobine Inhibits γ-Irradiation-Induced Cancer Cell Migration, Invasion and Metastasis in Non-Small Cell Lung Cancer Cells. Biomedicines.

[35] Lin P.H., Tseng C.L., Cheng Y.C., Ho C.H., Chen S.C., Wang Y., Liu E., Issafras H., Jiang W. (2021). Distinguishing features of a novel humanized anti-EGFR monoclonal antibody based on cetuximab with superior antitumor efficacy. Expert Opin. Biol. Ther..

[36] Huang H., Chen S., Van Doren J., Li D., Farichon C., He Y., Zhang Q., Zhang K., Conney A.H., Goodin S. (2015). α-Tomatine inhibits growth and induces apoptosis in HL-60 human myeloid leukemia cells. Mol. Med. Rep..

